# The polymorphism at residue 156 determines the HLA-B*35 restricted peptide repertoire during HCMV infection

**DOI:** 10.1007/s00251-018-1077-z

**Published:** 2018-08-21

**Authors:** Wiebke C. Abels, Trishna Manandhar, Heike Kunze-Schumacher, Rainer Blasczyk, Christina Bade-Döding

**Affiliations:** Institute for Transfusion Medicine, Hannover Medical School, Hannover, Germany

**Keywords:** HLA class I, HCMV, Antigen presentation, Peptides

## Abstract

Peptide selection in infected cells is not fully understood yet, but several indications point to the fact that there are differences to uninfected cells, especially in productive HCMV infection, since HCMV evolved various strategies to disable the hosts immune system, including presentation of peptide-HLA complexes to immune effector cells. Therefore, peptide predictions for specific HLA alleles are limited in these cases and the naturally presented peptide repertoire of HCMV-infected cells is of major interest to optimize adoptive T cell therapies. The allotypes HLA-B*35:01 and B*35:08 differ at a single amino acid at position 156 and have been described to differ in their peptide features and in their association with the peptide loading complex. Virus specific T cells recognizing the allelic pHLA-B*35 complexes could be detected, indicating a significant role of this HLA subtypes in viral immunity. However, naturally selected and presented viral peptides have not been described so far. In this study, we analyzed the peptide binding repertoire for HLA-B*35:01 and HLA-B*35:08 in HCMV-infected cells. The isolated peptides from both allelic subtypes were of extraordinary length, however differed in their features, origin, and sequence. For these HCMV-originated peptides, no overlap in the peptide repertoire could be observed between the two allelic subtypes. These findings reveal the discrepancies between predicted and naturally presented immunogenic epitopes and support the need of comprehensive peptide recruitment data for personalized and effective cellular therapies.

## Introduction

Human cytomegalovirus infections are widespread in the population. The prevalence varies depending on the country, the age and the socio-economic status, but is on average 40–90% (Cannon et al. 2010; De Paschale et al. 2010). Whereas healthy individuals show only weak or no symptoms at all, immunocompromised persons, e.g., after hematopoietic stem cell transplantation (HSCT), can develop severe complications. After primary infection, the virus establishes latency that alternate with short reactivation phases that are controlled by virus-specific cytotoxic T cells (CTLs) (Hahn et al. 1998; Mendelson et al. 1996; Taylor-Wiedeman et al. 1991).

Protection by CTLs is provided through recognition of viral peptides bound to HLA class I molecules (pHLA) (Sissons and Wills 2015). In the process, the specificity of the HLA allele determines which peptide is presented on the surface of an infected cell. The HLA genes are highly polymorphic with 5091 alleles for the HLA-B locus, resulting in 3664 different HLA-B proteins (Robinson et al. 2015). Some alleles differ in more than 30 amino acids (AA) from each other, while other differ only by one AA position. However, even a single AA difference that is not predicted to alter the peptide binding motif can result in a complete alteration of the peptide binding repertoire (Badrinath et al. 2014; Burrows et al. 2007; Manandhar et al. 2016). HLA-B*35:01 and HLA-B*35:08 differ only at position 156 with a 156Leu for B*35:01 and a 156Arg for B*35:08; their bound peptides possess different peptide binding characteristics even though AA 156 is not located at the primary peptide binding sites (B and F pocket), but protrudes from the α2-helix into the peptide binding groove (Huyton et al. 2012). The peptide binding motif is generally determined by the AAs in the pockets. Primary anchors for HLA-B*35:01 are Pro at position 2 (p2) and Tyr at the last position (pΩ) (and to a lesser extend Phe, Met, Leu, or Ile) (Falk et al. 1993; Hill et al. 1992; Manandhar et al. 2016; Schönbach et al. 1996). Additionally, secondary binding preferences are published for Leu, Val, Ile, or Met at p3 (Schönbach et al. 1996). The primary anchors for HLA-B*35:08 are also Pro at p2 and Tyr at pΩ (Manandhar et al. 2016). Burrows et al. (2007) also described Glu and Asp at p5 as potential secondary anchors that interact with 156Arg in B*35:08. The variation in the peptide binding repertoire that is created by a single AA difference provides a multifarious immune response across the population (Lawlor et al. 1990).

Since smallest structural alterations in the HLA molecule generated by a single AA substitution change the peptide repertoire significantly, while the primary peptide anchors remain unaltered, accurate prediction of peptide binding is exceedingly difficult. Even more challenging is peptide prediction for pathological situations, e.g., prediction of peptide presentation in virally infected cells. To date, this is, however, a common practice to define peptides that can be used for the stimulation and selection of T cells in adoptive T cell therapy (Sutrave et al. 2017).

Primary infections and especially reactivations of human cytomegalovirus (HCMV) after HSCT are frequent complications because of the impaired immune system and can lead to severe diseases (Afessa and Peters 2006; Ruell et al. 2007). Conventional antiviral drugs are still first-line therapy, but undesirable side effects and augmented presence of drug resistance gave rise to the development of other therapies (Fuji et al. 2017). In the adoptive T cell therapy, the knowledge of presented viral peptides is indispensable to identify virus specific CD8^+^ T cells. The usage of predicted peptides for the selection of appropriate T cells was already successful in several cases, but failed to have an effect in other cases (Doubrovina et al. 2012; Gottschalk et al. 2001; Pei et al. 2017; Schmitt et al. 2011; Withers et al. 2017). An obvious reason could be the inaccurate prediction of peptides. The triggering of T cells can be provoked for nearly every non-self-peptide bound to the particular HLA molecule, acknowledging the concept of the immune system, but these peptides have to be presented on the surface of infected cells in the respective patient for successful control of the viral infection. At that point, the HLA allele and the infected cell type may play the leading role accompanied by modulations of the peptide loading mechanism by the virus.

The present study describes previously unknown HCMV-derived peptides that are naturally presented by HCMV-infected fibroblasts on HLA-B*35:01 or HLA-B*35:08. Interestingly, these peptides do neither show the known peptide anchors nor the length of 8–11 AA that is proposed for HLA class I peptides.

## Materials and methods

### Cell culture and viruses

Fibroblast *BJ* cells (BJ, ATCC, Manassas, VA, USA) transduced with lentiviral constructs (exons 1–4) encoding for soluble (s)HLA-B*35:01 or (s)HLA-B*35:08 molecules were maintained in DMEM (Lonza, Basel, Switzerland) supplemented with 20% Medium 199 (Thermo Fisher Scientific Inc., Waltham, MA USA), 10% heat-inactivated fetal bovine serum (FBS; Lonza, Basel, Switzerland), and 2 mM L-glutamine (c-c Pro, Oberdorla, Germany). *HEK293T* cells were cultured in DMEM (Lonza, Basel, Switzerland) supplemented with 10% heat-inactivated FBS, 2 mM L-glutamine, and 1 mg/ml Geneticin® (Life Technologies, Carlsbad, USA). Primary fibroblast *NHDF-c* cells (PromoCell, Heidelberg, Germany) were maintained in DMEM supplemented with 10% heat-inactivated FBS and 2 mM L-glutamine. All cell lines were maintained at 37 °C and 5% CO_2_.

HCMV strain *AD169* was used for all infections and virus stock was prepared by infecting *NHDF-c* cells and pelleting the virus from cell supernatant at 100% cytopathic effect. The virus was stored at − 80 °C and titer was measured in plaque assay, performed on *NHDF-c* cells in carboxymethyl cellulose (CMC) medium.

### Engineering of soluble HLA molecules

For transduction, pRRL.PPT.SF.pre. V5-His/sHLA-B*35:01 and pRRL.PPT.SF.pre. V5-His/sHLA-B*35:08 were generated as previously described (Manandhar et al. 2016). The transduction of *BJ* cells was performed as detailed elsewhere (Kraemer et al. 2015). Quantitative analysis of sHLA molecules in the supernatant of transduced cells was performed via sandwich ELISA using the mAb w6/32 for coating and the anti-β2m pAb (Dako by Agilent Technologies, Santa Clara, USA) for detection.

### Large-scale production and purification of soluble HLA molecules

Transduced *BJ* cells were cultured in a two-compartment bioreactor for large-scale production of sHLA molecules. Cells in the bioreactor were infected with HCMV strain *AD169* at a multiplicity of infection (MOI) of 1 and sHLA containing supernatant was harvested weekly. Production was monitored by HLA-specific sandwich ELISA, and infectiosity was analyzed by infecting *BJ* cells with the supernatant collected from cells in the bioreactor and analyzed for cytopathic effect. Affinity purification was performed using a NHS- (N-hydroxysuccinimide-) activated HiTrap column precoupled to mAb W6/32. Bound trimeric complexes (HLA heavy chain, beta-2-microglobulin (β2m), peptide) were eluted with 0.1 M glycine/HCL buffer at pH 2.7. For further analysis, we used 2 mg purified trimeric complex of each allele.

### Identification of peptides via mass spectrometry

For the detection of peptides with different affinities, we utilized a technology for the identification of low and high binding peptides (Badrinath et al. 2012, 2014). The trimeric complexes were filtered postaffinity chromatography through an Amicon Ultra-15 Filter Unit (Merck, Darmstadt, Germany) with a 10-kD cutoff. Peptides detected in the flow-through were considered as low binding. The retentate was acidified with 0.1% trifluoroacetic acid and filtered again. Peptides in the acidified flow-through were defined as high binding. Peptide sequencing was performed utilizing the Eksigent Nano-LC Ultra 2D HPLC coupled to LTQ Orbitrap XL mass spectrometer (Thermo Fischer, Waltham, MA, USA). Peptides were identified using Mascot and the SwissProt human and HCMV database. This analysis has been repeated three times for reproducibility for each allele.

## Results

### HLA-B*35:01 and HLA-B*35:08 restricted peptide repertoires are divers during HCMV infection

To identify peptides that are presented by fibroblasts during HCMV infection, *BJ* cells were transduced with sHLA-B*35:01 or sHLA-B*35:08 constructs and infected with HCMV. sHLA molecules were purified from the cell culture supernatant, peptides eluted from the trimeric complexes and sequenced via mass spectrometry. During elution, the samples were divided into low- and high-affinity peptides.

For HLA-B*35:01, we found in total 156 different peptides, 2 peptides were derived from HCMV and 154 were of human origin (Table 1). In the low binding fraction, no HCMV-derived and only four human peptides could be identified. For the human (self-)peptides, there was no overlap between low and high binding peptides. In contrast, for HLA-B*35:08 a total of 260 peptides were identified. The four HCMV-derived peptides were all found in the high binding fraction, and again, for the self-peptides, the number of high binders (181) was higher than for low binders (75). Of 256 self-peptides, 23 were present in the low and in the high binding fraction. All other peptides were exclusively in the respective fraction.

**Table 1:** Number of identified peptides in the human or HCMV database specific for HLA-B*35:01 or HLA-B*35:08

HLA allele and species in database	LB/HB peptides	Count
B*35:01—human	LB: 4 HB: 150	154
B*35:01—HCMV	LB: 0 HB: 2	2
B*35:08—human	LB: 75 HB: 181	256, with 23 in LB and HB
B*35:08—HCMV	LB: 0 HB: 4	4

*HB* high binding, *LB* low binding

Notably, between HLA-B*35:01 and HLA-B*35:08 was no overlap of HCMV-derived peptides. Self-peptides found in the human database showed an overlap of 53 peptides.

### HCMV infection alters the peptide binding motif

**Fig. 1:**
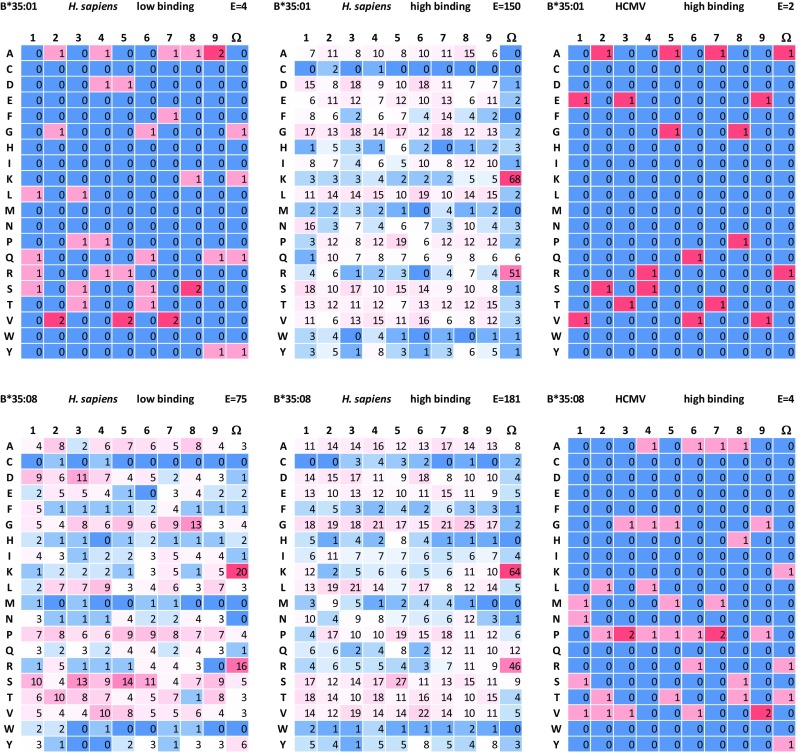
Preferred aa at different positions within the peptide. For HLA-B*35:01 and HLA-B*35:08, the preferred aa in p1–9 and pΩ were determined separately for human- and HCMV-derived peptides and for low and high binding peptides. Peptides that were present more frequently than once in one condition were counted only once. Color coded is the number of the aa at that position with red the highest and blue the lowest numbers

The published peptide binding motif for HLA-B*35:01 and HLA-B*35:08 is the same: Pro at peptide position (p)2 and Tyr at pΩ (Falk et al. 1993; Hill et al. 1992; Manandhar et al. 2016). These peptide anchors are determined in uninfected cells. In HCMV-infected fibroblasts, we could observe different peptide binding motifs for HLA-B*35:01 as well as for HLA-B*35:08 (Fig. 1). For HLA-B*35:01, the number of human-derived low binding and for both alleles the number of HCMV-derived peptides is too low to make a statement about the binding motif. Peptides from the other HCMV conditions in fibroblasts (HLA-B*35:01 high binding and HLA-B*35:08 low and high binding) show a strong preference for Lys and Arg.

### Human peptides from infected cells show a broad length distribution

**Fig. 2:**
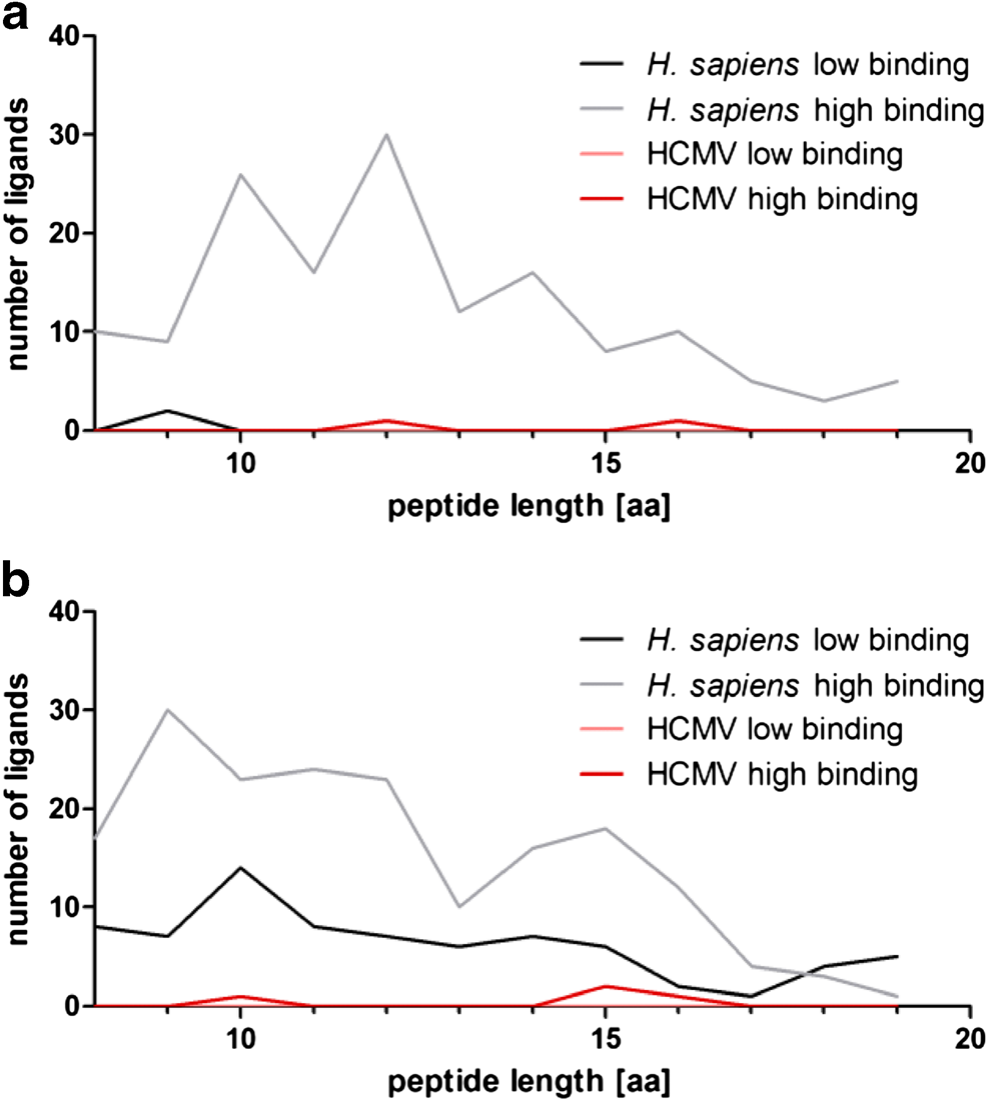
Length distribution of human- and HCMV-derived peptides. **a** Low and high binding peptides isolated from HCMV-infected BJ/HLA-B*35:01 cells. **b** Low and high binding peptides isolated from HCMV-infected BJ/HLA-B*35:08 cells

Regarding the length of the identified peptides, small differences between HLA-B*35:01 and HLA-B*35:08 became apparent (Fig. 2). High binding HLA-B*35:01 self-peptides show peaks for 10 and 12 AA length. High binding self-peptides derived from HLA-B*35:08 show a broader length distribution with a small peak for 9 AAs. Low binding peptides from the same allele are widely distributed over different length as well. A small peak is at a length of 10 AAs.

HCMV-derived peptides presented on infected fibroblasts vary in their length as well. HLA-B*35:01-restricted HCMV peptides are 12 and 16 AAs long, and HLA-B*35:08-restricted HCMV peptides featured a length of 10, 15, and 16 AAs.

### The isolated HCMV peptides are derived from different proteins

**Fig. 3:**
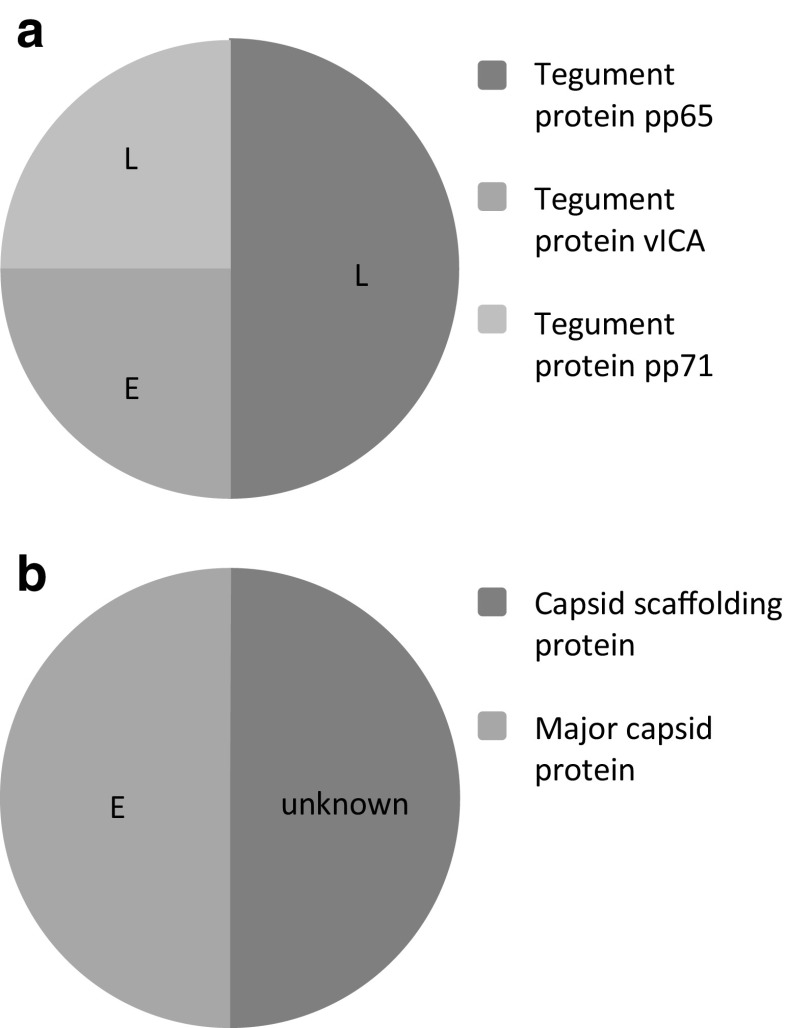
Protein origin of HCMV-derived peptides. Indicated is also the expression time point of the respective protein in the lytic replication cycle. *E* proteins from the early phase, *L* proteins from the late phase. **a** Peptides isolated from HCMV-infected BJ/HLA-B*35:01 cells. **b** Peptides isolated from HCMV-infected BJ/HLA-B*35:08 cells

HCMV-derived peptides from HLA-B*35:01 (Table 2) and HLA-B*35:08 (Table 3) originate from different proteins and different time points of the infection stage (Fig. 3). The two HLA-B*35:01 specific peptides are from the capsid scaffolding protein and the major capsid protein. Both proteins are relevant for the production of new viral particles. Genes responsible for this step in the lytic replication cycle are rather later transcribed genes. The major capsid protein is characterized as an early (E)–late (L) protein (Chambers et al. 1999), whereas the time point for the capsid scaffolding protein is unknown, but presumably it is the same period. We found four HLA-B*35:08 specific peptides from three different HCMV proteins: one from the tegument proteins pp71 and vICA each and two from the tegument protein pp65. All three proteins are involved in immune evasion mechanisms. Pp65 and pp71 are both responsible for the suppression of the presentation of peptide-HLA complexes (Tomtishen 2012). Additionally, pp65 can inhibit NK cell reactions and pp71 is required for efficient activation of immediate-early (IE) genes in the lytic replication cycle. The third protein, vICA, is a caspase 8 inhibitor that prevents Fas-induced apoptosis (Skaletskaya et al. 2001).

**Table 2:** HCMV-derived HLA-B*35:01 specific peptides

Sequence	Length	Source
VAERAQAGVVNA	12	HCMVA capsid scaffolding protein
ESTSGVTPEDSIAAQR	16	HCMV major capsid protein

**Table 3:** HCMV-derived HLA-B*35:08 specific peptides

Sequence	Length	Source
MPPLTPPHVY	10	HCMV tegument protein vICA
SVPAPRPSPISTAST	15	HCMV tegument protein pp71
NLVPMVATVQGQNLK	15	HCMV tegument protein pp65
VTGGGAMAGASTSAGR	16	HCMV tegument protein pp65

## Discussion

Since binding of short peptides to HLA class I molecules was shown over 30 years ago (Bjorkman et al. 1987a; Bjorkman et al. 1987b), it is widely accepted that class I molecules bind only 8–11 AA long peptides. Several studies showed that this assumption holds true for most ligands (Badrinath et al. 2014; Huczko et al. 1993; Hunt et al. 1992; Manandhar et al. 2016; Rammensee et al. 1993). The theory that only peptides of 8–11 AAs are bound is underlined by the structure of HLA class I molecules, where the peptide binding region (PBR) is divided in six specificity pockets and seems to restrict the length of bound ligands to 8–11 AAs. However, in the last years, it became more and more evident that longer peptides can be selected and presented as well (Bade-Doding et al. 2011; Bell et al. 2009; Burrows et al. 2006; McMurtrey et al. 2016; Remesh et al. 2017). Several investigations revealed the mechanisms by which long peptides can be bound in the PBR: (I) peptides are bound at the N- and C-termini in the peptide binding pockets and the middle part that is too long to fit in the binding cleft bulges out (Probst-Kepper et al. 2004; Stewart-Jones et al. 2005; Tynan et al. 2005a; Wynn et al. 2008) and (II) the C-terminal part of the peptide can project into the solvent by opening the F pocket (McMurtrey et al. 2016; Remesh et al. 2017). The first expectation that the conformation of these peptides will not be recognized by T cell receptors (Guo et al. 1992) could be disproved. Tynan et al. (2005b) demonstrated that the T cell receptor makes minimal contacts to HLA and recognizes the bulged peptide in an antibody-like manner.

It was suggested by Bell et al. (2009) that the length restriction of presented peptides depends in most cases less on the HLA allele, but on the availability of peptides and can include peptides with up to 25 AAs, especially, in HLA alleles that are strictly anchored at the N- and C-termini (p2 and pΩ) as HLA-A*24:02, HLA-B*07:02, and HLA-B*35:01. Particularly in the condition of an infection, like CMV infection, the availability of peptides might be changed compared to uninfected cells because of the multiple interactions of the virus with structures involved in antigen presentation (Ahn et al. 1997; Furman et al. 2002; Halenius et al. 2011; Jones et al. 1995; Jones and Sun 1997; Jones et al. 1996; Kim et al. 2008). Our presented investigations are in concordance with these findings and suggestions. We assume that changes in the peptide anchors observed in infected cells are also driven by the disturbed peptide loading and presentation process. Interestingly, the preferred AAs Lys and Arg at pΩ are the same in HLA-B*35:01 and HLA-B*35:08 as described for uninfected cells (Manandhar et al. 2016).

However, our previous studies indicated the functional difference between HLA-B*35:01 and HLA-B*35:08. Even between peptides acquired by these B*35 variants in the same cell no overlap in the peptide repertoire from more than 10% could be observed.

A striking difference in the interaction with the peptide loading complex (PLC) could be described for the B*35 subtypes. The AA difference at residue 156 impacts significantly the association with tapasin (TPN) and TAP; while HLA-B*35:01 proved to recruit peptides more TPN-independent, B*35:08 recruited peptides in a more TAP-independent manner (Manandhar et al. 2016). These findings suggest that both subtypes would select and present a different set of peptides during an infection when the function of certain PLC components are temporary disrupted. Besides general information on peptide binding modalities during infection, identification of HCMV-derived peptides that are naturally presented by infected cells is a crucial task in the development of effective adoptive T cell therapies that could, to our knowledge, not be fulfilled yet. In this study, we present two HCMV-derived peptides specific for HLA-B*35:01 and four specific for HLA-B*35:08. No overlap in the bound HCMV peptides could be observed, despite both alleles share the same preferred AAs. These results fit to observations that the single mismatch at AA 156 can modulate peptide binding, peptide conformation, peptide flexibility, and CTL responses in different alleles (Burrows et al. 2007; Miles et al. 2006; Tynan et al. 2005c). Burrows et al. (2007) demonstrated that differences in the peptide binding repertoire of HLA-B*35:01 and HLA-B*35:08 originate from different dissociation kinetics.

HLA-B*35:08 restricted HCMV-derived peptides are all derived from proteins that play a role in the viral immune evasion. This observation is of major interest, since the proteins are essential for efficient hiding and viral mutation in one of these proteins can lead to reduced immune evasion capacity. Additionally, the source proteins for these HCMV peptides are expressed at different time points of the lytic infection cycle and could broaden the peptide repertoire for T cell therapies.

The selection of viral peptides suitable for adoptive T cell therapies is based on peptides of 8–11-aa length. These peptides have never been proven to be naturally presented by HLA molecules. We provide evidence that HLA-B*35:01/08 molecules can naturally select and present HCMV-derived peptides that (i) exhibit an extraordinary length and (ii) do not match the peptide binding motifs; hence, the concept for peptide prediction should be rethought.

## References

[CR1] Afessa B, Peters SG (2006). Major complications following hematopoietic stem cell transplantation. Semin Respir Crit Care Med.

[CR2] Ahn K, Gruhler A, Galocha B, Jones TR, Wiertz EJ, Ploegh HL, Peterson PA, Yang Y, Früh K (1997). The ER-luminal domain of the HCMV glycoprotein US6 inhibits peptide translocation by TAP. Immunity.

[CR3] Bade-Doding C, Theodossis A, Gras S, Kjer-Nielsen L, Eiz-Vesper B, Seltsam A, Huyton T, Rossjohn J, McCluskey J, Blasczyk R (2011). The impact of human leukocyte antigen (HLA) micropolymorphism on ligand specificity within the HLA-B*41 allotypic family. Haematologica.

[CR4] Badrinath S, Saunders P, Huyton T, Aufderbeck S, Hiller O, Blasczyk R, Bade-Doeding C (2012). Position 156 influences the peptide repertoire and tapasin dependency of human leukocyte antigen B*44 allotypes. Haematologica.

[CR5] Badrinath S, Kunze-Schumacher H, Blasczyk R, Huyton T, Bade-Doeding C (2014). A micropolymorphism altering the residue triad 97/114/156 determines the relative levels of tapasin independence and distinct peptide profiles for HLA-A(*)24 allotypes. J Immunol Res.

[CR6] Bell MJ, Burrows JM, Brennan R, Miles JJ, Tellam J, McCluskey J, Rossjohn J, Khanna R, Burrows SR (2009). The peptide length specificity of some HLA class I alleles is very broad and includes peptides of up to 25 amino acids in length. Mol Immunol.

[CR7] Bjorkman PJ, Saper MA, Samraoui B, Bennett WS, Strominger JL, Wiley DC (1987). The foreign antigen binding site and T cell recognition regions of class I histocompatibility antigens. Nature.

[CR8] Bjorkman PJ, Saper MA, Samraoui B, Bennett WS, Strominger JL, Wiley DC (1987). Structure of the human class I histocompatibility antigen, HLA-A2. Nature.

[CR9] Burrows SR, Rossjohn J, McCluskey J (2006). Have we cut ourselves too short in mapping CTL epitopes?. Trends Immunol.

[CR10] Burrows JM, Wynn KK, Tynan FE, Archbold J, Miles JJ, Bell MJ, Brennan RM, Walker S, McCluskey J, Rossjohn J, Khanna R, Burrows SR (2007). The impact of HLA-B micropolymorphism outside primary peptide anchor pockets on the CTL response to CMV. Eur J Immunol.

[CR11] Cannon MJ, Schmid DS, Hyde TB (2010). Review of cytomegalovirus seroprevalence and demographic characteristics associated with infection. Rev Med Virol.

[CR12] Chambers J, Angulo A, Amaratunga D, Guo H, Jiang Y, Wan JS, Bittner A, Frueh K, Jackson MR, Peterson PA, Erlander MG, Ghazal P (1999). DNA microarrays of the complex human cytomegalovirus genome: profiling kinetic class with drug sensitivity of viral gene expression. J Virol.

[CR13] De Paschale M, Agrappi C, Manco MT, Clerici P (2010). Positive predictive value of anti-HCMV IgM as an index of primary infection. J Virol Methods.

[CR14] Doubrovina E, Oflaz-Sozmen B, Prockop SE, Kernan NA, Abramson S, Teruya-Feldstein J, Hedvat C, Chou JF, Heller G, Barker JN, Boulad F, Castro-Malaspina H, George D, Jakubowski A, Koehne G, Papadopoulos EB, Scaradavou A, Small TN, Khalaf R, Young JW, O'Reilly RJ (2012). Adoptive immunotherapy with unselected or EBV-specific T cells for biopsy-proven EBV+ lymphomas after allogeneic hematopoietic cell transplantation. Blood.

[CR15] Falk K, Rötzschke O, Grahovac B, Schendel D, Stevanović S, Jung G, Rammensee HG (1993). Peptide motifs of HLA-B35 and -B37 molecules. Immunogenetics.

[CR16] Fuji S, Einsele H, Kapp M (2017). Cytomegalovirus disease in hematopoietic stem cell transplant patients: current and future therapeutic options. Curr Opin Infect Dis.

[CR17] Furman MH, Dey N, Tortorella D, Ploegh HL (2002). The human cytomegalovirus US10 gene product delays trafficking of major histocompatibility complex class I molecules. J Virol.

[CR18] Gottschalk S, Ng CYC, Perez M, Smith CA, Sample C, Brenner MK, Heslop HE, Rooney CM (2001). An Epstein-Barr virus deletion mutant associated with fatal lymphoproliferative disease unresponsive to therapy with virus-specific CTLs. Blood.

[CR19] Guo H-C, Jardetzky TS, Garrettt TPJ, Lane WS, Strominger JL, Wiley DC (1992). Different length peptides bind to HLA-Aw68 similarly at their ends but bulge out in the middle. Nature.

[CR20] Hahn G, Jores R, Mocarski ES (1998). Cytomegalovirus remains latent in a common precursor of dendritic and myeloid cells. Proc Natl Acad Sci U S A.

[CR21] Halenius A, Hauka S, Dolken L, Stindt J, Reinhard H, Wiek C, Hanenberg H, Koszinowski UH, Momburg F, Hengel H (2011). Human cytomegalovirus disrupts the major histocompatibility complex class I peptide-loading complex and inhibits tapasin gene transcription. J Virol.

[CR22] Hill AV, Elvin J, Willis AC, Aidoo M, Allsopp CE, Gotch FM, Gao XM, Takiguchi M, Greenwood BM, Townsend AR, McMichael AJ, Whittle HC (1992). Molecular analysis of the association of HLA-B53 and resistance to severe malaria. Nature.

[CR23] Huczko EL, Bodnar WM, Benjamin D, Sakaguchi K, Zhu N, Shabanowitz J, Henderson RA, Appella E, Hunt DF, Engelhard VH (1993). Characteristics of endogenous peptides eluted from the class I MHC molecule HLA-B7 determined by mass spectrometry and computer modeling. J Immunol.

[CR24] Hunt DF, Henderson RA, Shabanowitz J, Sakaguchi K, Michel H, Sevilir N, Cox AL, Appella E, Engelhard VH (1992). Characterization of peptides bound to the class I MHC molecule HLA-A2.1 by mass spectrometry. Science.

[CR25] Huyton T, Ladas N, Schumacher H, Blasczyk R, Bade-Doeding C (2012). Pocketcheck: updating the HLA class I peptide specificity roadmap. Tissue Antigens.

[CR26] Jones TR, Sun L (1997). Human cytomegalovirus US2 destabilizes major histocompatibility complex class I heavy chains. J Virol.

[CR27] Jones TR, Hanson LK, Sun L, Slater JS, Stenberg RM, Campbell AE (1995). Multiple independent loci within the human cytomegalovirus unique short region down-regulate expression of major histocompatibility complex class I heavy chains. J Virol.

[CR28] Jones TR, Wiertz EJ, Sun L, Fish KN, Nelson JA, Ploegh HL (1996). Human cytomegalovirus US3 impairs transport and maturation of major histocompatibility complex class I heavy chains. Proc Natl Acad Sci U S A.

[CR29] Kim Y, Park B, Cho S, Shin J, Cho K, Jun Y, Ahn K (2008). Human cytomegalovirus UL18 utilizes US6 for evading the NK and T-cell responses. PLoS Pathog.

[CR30] Kraemer T, Celik AA, Huyton T, Kunze-Schumacher H, Blasczyk R, Bade-Doding C (2015). HLA-E: presentation of a broader peptide repertoire impacts the cellular immune response-implications on HSCT outcome. Stem Cells Int.

[CR31] Lawlor DA, Zemmour J, Ennis PD, Parham P (1990). EVOLUTION OF CLASS-I MHC GENES AND PROTEINS: from natural selection to Thymic selection. Annu Rev Immunol.

[CR32] Manandhar T, Kunze-Schumacher H, Huyton T, Celik AA, Blasczyk R, Bade-Doeding C (2016). Understanding the obstacle of incompatibility at residue 156 within HLA-B*35 subtypes. Immunogenetics.

[CR33] McMurtrey C, Trolle T, Sansom T, Remesh SG, Kaever T, Bardet W, Jackson K, McLeod R, Sette A, Nielsen M, Zajonc DM, Blader IJ, Peters B, Hildebrand W (2016) Toxoplasma gondii peptide ligands open the gate of the HLA class I binding groove. Elife 5 10.7554/eLife.12556 PMC4775218 26824387

[CR34] Mendelson M, Monard S, Sissons P, Sinclair J (1996). Detection of endogenous human cytomegalovirus in CD34+ bone marrow progenitors. J Gen Virol.

[CR35] Miles JJ, Borg NA, Brennan RM, Tynan FE, Kjer-Nielsen L, Silins SL, Bell MJ, Burrows JM, McCluskey J, Rossjohn J, Burrows SR (2006). TCR genes direct MHC restriction in the potent human T cell response to a class I-bound viral epitope. J Immunol.

[CR36] Pei XY, Zhao XY, Chang YJ, Liu J, Xu LP, Wang Y, Zhang XH, Han W, Chen YH, Huang XJ (2017). Cytomegalovirus-specific T-cell transfer for refractory cytomegalovirus infection after Haploidentical stem cell transplantation: the quantitative and qualitative immune recovery for cytomegalovirus. J Infect Dis.

[CR37] Probst-Kepper M, Hecht HJ, Herrmann H, Janke V, Ocklenburg F, Klempnauer J, van den Eynde BJ, Weiss S (2004). Conformational restraints and flexibility of 14-Meric peptides in complex with HLA-B*3501. J Immunol.

[CR38] Rammensee HG, Falk K, Rötzschke O (1993). Peptides naturally presented by MHC class I molecules. Annu Rev Immunol.

[CR39] Remesh SG, Andreatta M, Ying G, Kaever T, Nielsen M, McMurtrey C, Hildebrand W, Peters B, Zajonc DM (2017). Unconventional peptide presentation by major histocompatibility complex (MHC) class I allele HLA-A*02:01: BREAKING CONFINEMENT. J Biol Chem.

[CR40] Robinson J, Halliwell JA, Hayhurst JD, Flicek P, Parham P, Marsh SG (2015). The IPD and IMGT/HLA database: allele variant databases. Nucleic Acids Res.

[CR41] Ruell J, Barnes C, Mutton K, Foulkes B, Chang J, Cavet J, Guiver M, Menasce L, Dougal M, Chopra R (2007). Active CMV disease does not always correlate with viral load detection. Bone Marrow Transplant.

[CR42] Schmitt A, Tonn T, Busch DH, Grigoleit GU, Einsele H, Odendahl M, Germeroth L, Ringhoffer M, Ringhoffer S, Wiesneth M, Greiner J, Michel D, Mertens T, Rojewski M, Marx M, von Harsdorf S, Dohner H, Seifried E, Bunjes D, Schmitt M (2011). Adoptive transfer and selective reconstitution of streptamer-selected cytomegalovirus-specific CD8+ T cells leads to virus clearance in patients after allogeneic peripheral blood stem cell transplantation. Transfusion.

[CR43] Schönbach C, Miwa K, Ibe M, Shiga H, Nokihara K, Takiguchi M (1996). Refined peptide HLA-B*3501 binding motif reveals differences in 9-mer to 11-mer peptide binding. Immunogenetics.

[CR44] Sissons JG, Wills MR (2015). How understanding immunology contributes to managing CMV disease in immunosuppressed patients: now and in future. Med Microbiol Immunol.

[CR45] Skaletskaya A, Bartle LM, Chittenden T, McCormick AL, Mocarski ES, Goldmacher VS (2001). A cytomegalovirus-encoded inhibitor of apoptosis that suppresses caspase-8 activation. Proc Natl Acad Sci U S A.

[CR46] Stewart-Jones GBE, Gillespie G, Overton IM, Kaul R, Roche P, McMichael AJ, Rowland-Jones S, Jones EY (2005). Structures of three HIV-1 HLA-B*5703-peptide complexes and identification of related HLAs potentially associated with long-term nonprogression. J Immunol.

[CR47] Sutrave G, Blyth E, Gottlieb DJ (2017). Cellular therapy for multiple pathogen infections after hematopoietic stem cell transplant. Cytotherapy.

[CR48] Taylor-Wiedeman J, Sissons JGP, Borysiewicz LK, Sinclair JH (1991). Monocytes are a major site of persistence of human cytomegalovirus in peripheral blood mononuclear cells. J Gen Virol.

[CR49] Tomtishen JP (2012). Human cytomegalovirus tegument proteins (pp65, pp71, pp150, pp28). Virol J.

[CR50] Tynan FE, Borg NA, Miles JJ, Beddoe T, El-Hassen D, Silins SL, van Zuylen WJ, Purcell AW, Kjer-Nielsen L, McCluskey J, Burrows SR, Rossjohn J (2005). High resolution structures of highly bulged viral epitopes bound to major histocompatibility complex class I. Implications for T-cell receptor engagement and T-cell immunodominance. J Biol Chem.

[CR51] Tynan FE, Burrows SR, Buckle AM, Clements CS, Borg NA, Miles JJ, Beddoe T, Whisstock JC, Wilce MC, Silins SL, Burrows JM, Kjer-Nielsen L, Kostenko L, Purcell AW, McCluskey J, Rossjohn J (2005). T cell receptor recognition of a 'super-bulged' major histocompatibility complex class I-bound peptide. Nat Immunol.

[CR52] Tynan FE, Elhassen D, Purcell AW, Burrows JM, Borg NA, Miles JJ, Williamson NA, Green KJ, Tellam J, Kjer-Nielsen L, McCluskey J, Rossjohn J, Burrows SR (2005). The immunogenicity of a viral cytotoxic T cell epitope is controlled by its MHC-bound conformation. J Exp Med.

[CR53] Withers B, Blyth E, Clancy LE, Yong A, Fraser C, Burgess J, Simms R, Brown R, Kliman D, Dubosq M-C, Bishop D, Sutrave G, Ma CKK, Shaw PJ, Micklethwaite KP, Gottlieb DJ (2017). Long-term control of recurrent or refractory viral infections after allogeneic HSCT with third-party virus-specific T cells. Blood Adv.

[CR54] Wynn KK, Fulton Z, Cooper L, Silins SL, Gras S, Archbold JK, Tynan FE, Miles JJ, McCluskey J, Burrows SR, Rossjohn J, Khanna R (2008). Impact of clonal competition for peptide-MHC complexes on the CD8+ T-cell repertoire selection in a persistent viral infection. Blood.

